# Optimization of Capillary Vibrating Sharp-Edge Spray Ionization for Native Mass Spectrometry of Triplex DNA

**DOI:** 10.1021/acsomega.4c10615

**Published:** 2025-03-27

**Authors:** Sultan Mahmud, Vikum K. Dewasurendra, Chandrima Banerjee, Pedram Tavadze, Mst Nigar Sultana, Mohammad A. Rahman, Sohag Ahmed, Peng Li, Matthew B. Johnson, Stephen J. Valentine

**Affiliations:** † Department of Chemistry, West Virginia University, Morgantown, West Virginia 26506, United States; ‡ Department of Physics and Astronomy, West Virginia University, Morgantown, West Virginia 26506, United States

## Abstract

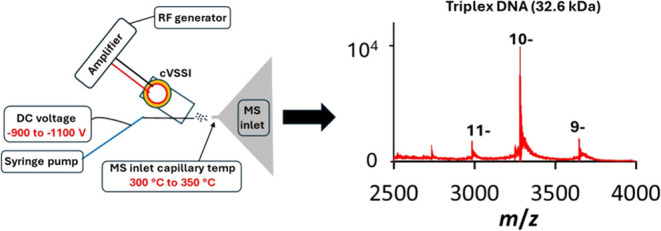

Capillary vibrating sharp-edge spray ionization (cVSSI) has been used to study the effects of applied voltage and mass spectrometer heated inlet transfer tube temperature on DNA triplex ion production for native mass spectrometry (MS) samples. Overall, medium applied voltage (−900 to −1000 V) results in better ion production of the desired triplex ions (Tri) (i.e., those without cation adducts such as NH_4_^+^, Na^+^, and K^+^); mass spectral peak intensities for the [Tri]^8–^, [Tri]^9–^, and [Tri]^10–^ ions increase by ∼70, ∼260, and ∼125 fold, respectively, compared to higher voltages (−1100 to −1500 V). The latter voltages result in increased triplex adduct ion (Tri + ad) formation; for the 8–, 9–, and 10– charge states; the ratios of Tri to Tri+ad ion abundances increase by ∼6 fold for the lower voltage. By capillary inlet temperatures of 300 to 400 °C, Tri ion abundances reach maximum values of 6.1 × 10^5^ ([Tri]^8–^), 2.9 × 10^6^ ([Tri]^9–^), and 6.4 × 10^5^ ([Tri]^10–^). Ion abundances for the respective species decrease by ∼4, ∼14, and ∼190 fold at a heated inlet transfer tube temperature of 450 °C. The abundances for Tri+ad ions species generally follow a similar trend as a function of heated inlet transfer tube temperature with the exception that maximum values are obtained at 250 °C. The abundances for DNA triplex fragment ions (Tri-fr) reach maximum values at 400 °C resulting from excessive, in-source ion activation. From these studies, the optimal capillary MS inlet temperature for production of large oligonucleotides by cVSSI is 300 to 350 °C and the applied voltage should be maintained at ∼ −900 V. These studies lay the foundation for native MS of large oligonucleotide species in negative-ion mode exploiting the sensitivity enhancements of cVSSI.

## Introduction

Native mass spectrometry (MS)^1−4^ is an established yet rapidly developing tool for the structural analysis of various biomolecules characterized under pseudophysiological conditions.^5−14^ Development in native MS coincides with the study of functional oligonucleotides as therapeutic targets/agents, diagnostic tools, expression control agents, and nanomaterial sources.^15−22^ Because of the sensitivity of MS and the ability of native MS to provide supplementary structural information, it has found increasing use in the study of functional oligonucleotides.^23−37^

One challenge in the study of oligonucleotide species by native MS is that for electrospray ionization (ESI)^38^ and nanoelectrospray ionization (nESI)^39,40^ the corona discharge potential under ambient conditions is near the onset of formation of the Taylor cone leading to lowered sensitivity.^41−44^ Although workarounds such as the use of pressurized ion sources,^45^ high impedance voltage application,^46,47^ and nondirect droplet plume introduction strategies (e.g., desorption electrospray ionization, DESI, and Single-probe MSI)^44^ have been introduced, often these require source modifications that may hinder proliferation of native MS applications and instrumentation. One limitation of oligonucleotide native MS is that functional structures often require high concentrations of metal cations,^48−50^ which dramatically reduces the overall sensitivity of MS measurements. Therefore, strategies that improve native MS sensitivity in negative-ion mode are desired for functional oligonucleotide structure characterization.^51^ Although nanoscale emitter tips are shown to effectively mitigate high salt concentrations,^52^ for negative-ion mode, nESI implemented in this manner is still subject to corona discharge concerns.^53^

Several field-free ionization methodologies have been examined for their ability to characterize biopolymers. Inlet ionization^54^ and vibrating tip spray ionization^55^ have been demonstrated to provide ESI-like charge state distributions (CSDs) for peptides and proteins. More recently, field-free spray techniques have been examined for native MS applications. Standing-wave surface acoustic wave nebulization (SW-SAWN) has been used to study protein analytes by native MS.^56^ The observation of very low CSDs and dimer ions, indicative of native structure preservation, was suggestive of an ionization process that is gentler than ESI. Shortly thereafter, the technique of mechanospray ionization (MoSI) was introduced.^57^ Again, very low CSDs for different protein samples resulted from MoSI-MS. Remarkably, in some cases, a second, lower CSD was observed suggesting the observation of native protein conformers not accessible by ESI. Capillary vibrating sharp-edge spray ionization (cVSSI)^58,59^ was employed to examine globular proteins from native MS solutions.^60^ cVSSI also showed an ability to better preserve the structures of fragile proteins that undergo partial denaturation with the application of voltages used in ESI and nESI.

Experiments have shown that combining the cVSSI process with the application of a DC voltage to the infused solution provides superior ionization, compared with ESI, in negative-ion mode for a variety of molecular species.^51,61^ However, recent work has shown that different voltages applied to the cVSSI emitter tip can result in different pathways to ion production.^62^ At very low voltages, native structure is preserved. As the voltage is increased, biopolymer structure is not preserved. At the highest voltages, native structure is again preserved. Such effects are proposed to result from different processes that produce the progeny nanodroplets from which the ions ultimately emerge. For example, at zero volts and very low voltages, it is likely that large droplets undergo aerodynamic breakup leading to progeny nanodroplet charge separation and production of species of very low charge and preservation of fragile biopolymer structure.^62^ That said, high-voltage conditions produce the greatest number of ions. Therefore, to take full advantage of cVSSI for native MS experiments of functional oligonucleotides, it is necessary to map experimental conditions that provide the highest sensitivities while preserving structure.

In this study, two ion source parameters are investigated for their ability to produce the desired [M–*n*H]^*n*−^ ions (i.e., those containing no adduct cations such as NH_4_^+^, Na^+^, and K^+^) in native MS experiments for DNA triplex molecules. The two parameters examined are DC voltage applied between the tip and inlet, and heated inlet transfer tube temperature. Overall, the highest efficiency ion production for the desired triplex species exhibiting low charge state is determined to be ∼ −900 V and the optimal heated inlet transfer tube temperature is determined to fall between 300 and 350 °C. The selection of triplex DNA is based on its potential for future therapeutics and the foundational native MS work showing structure preservation by ESI-MS.^63,64^

## Methods and Materials

### Chemicals and Reagents

The single strands (GAA)_12_ and (TTC)_12_ (for 36mer Triplex) and (GAA)_9_ and (TTC)_9_ (for 27mer Triplex) DNA oligonucleotides were purchased from Integrated DNA Technologies, Inc. HPLC grade water, nuclease free water, and ammonium acetate were purchased from Fisher Scientific. All chemicals and solvents were used without further purification.

### Sample Preparation

DNA molecules were dissolved in nuclease free water to prepare a 100 μM stock solution. One molar ammonium acetate buffer solution was prepared in nuclease free water. The final concentration in native MS samples was 50 μM triplex DNA in 400 mM ammonium acetate buffer, where the (GAA)_*x*_ and (TTC)_*x*_ (*x* = 9 for 27-mer DNA, and *x* = 12 for 36-mer DNA) were added in a 1:2 molar ratio. The excess amount of (TTC)_*x*_ acts as the third strand, or triplex forming oligonucleotide (TFO), which binds in the major groove of duplex DNA. The pH of the solution was set to 5.5, which enhances structural stability of the triplex DNA.^23^ Triplex DNA formation resulted from an annealing process that involves increasing the solution temperature to 90 °C and maintaining the temperature for 10 min, then slowly cooling it to room temperature. The annealed sample was refrigerated at 4 °C for 24 h prior to MS experiments.

### cVSSI Device Fabrication

A schematic of the cVSSI device is shown in Figure S1. Briefly, capillary segments (glass precision capillary tube, 400 μm I.D., Drummond Scientific) were pulled using a micropipette puller (P-2000, Sutter Instrument). The I.D. of the pulled capillary emitter tips typically range from 15 to 30 μm. The emitter tip was attached to one end of a microscope glass slide (24 × 60 mm^2^, #1 VWR) using glass glue (Figure S1). A piezoelectric transducer (4.6 kHz, 7BB-27–4LO, Murata) was attached to the other end of the glass slide with epoxy glue. The cVSSI devices were maintained at room temperature for 3 days for curing. Radio frequency voltage (10 V_pp_ at ∼95 kHz) was applied to the devices using a function generator (RIGOL DG-quadruplex102) and a power amplifier (Krohn-Hite 7500). One end of a PTFE tube (30-gauge thin-wall tubing, Cole-Parmer) was connected to the back of the capillary and the other end was connected to the sample syringe. Each device was tested for droplet plume production using an appropriate flow rate (2 to 5 μL/min) provided by a syringe pump (Fusion 200, Chemyx). For each device, the frequency was first optimized by changing the frequency by 200 Hz increments at a set voltage of 9 Vpp. The plume was visually monitored to observe the plume production characterized by a smoke-like mist. Next the voltage was changed by 1 Vpp across a range of 8 to 12 Vpp to optimize the plume production at the set frequency. A ∼7 cm-long Pt wire was inserted into the PTFE tube by puncturing it with a needle and sealing the tiny hole with epoxy glue to achieve good electrical contact. This connection was made near the capillary emitter tip junction (see Figure S1). A DC bias voltage was provided to the Pt wire using a high-voltage power supply (HEOPS-10B2, Matsusada Precision) The sample flow rate for all experiments was maintained at 2 μL/min. For the temperature-dependent studies, a single cVSSI device was utilized.

### MS Settings

Mass spectrometric measurements of the triplex DNA species were performed on a Q-Exactive hybrid quadrupole-orbitrap mass spectrometer (Thermo Fisher Scientific) under negative-ion mode. The full spectral scan range was *m*/*z* 1500 to 4000. For all experiments a resolving power of 140,000 was used except for the first MS scans conducted at 250 °C where a resolving power of 70,000 was used. For subsequent temperature settings the resolving power was increased to fully distinguish isotopologue triplex ion features. The maximum injection time was maintained at 1000 ms and an automatic gain control (AGC) setting of 1 × 10^6^ was used. To enhance the mass spectral peaks associated with the triplex ions not containing adducts, in-source activation was applied at 30 eV. For the temperature-dependent studies of DNA triplex, 7 different heated inlet transfer tube temperatures (150, 200, 250, 300, 350, 400, and 450 °C) were used. For the voltage-dependent study, the DC voltage applied to the solution ranged from 0 to −1,600 to 0 V using a 100 V step size at all temperature settings.

### Data Processing

MS. RAW data were exported as excel files using a Python script. To generate a heat map of the mass spectral data, a rectangular matrix with constant mass-to-charge ratio (*m*/*z*) spacing and (slightly) different time intervals was generated using MATLAB. Linear interpolation for evenly spaced *m*/*z* is performed because the *m*/*z* bins in the. RAW files are not evenly spaced, nor the same for each spectrum (irregular matrix or variable number of entries in each row). This was used for all MS spectral averaging, heat map generation, etc. Start times for each spectrum are not evenly spaced as well, due to AGC use. We do not interpolate the start times. Mass spectral scans were synchronized to applied voltage using the timing from a video recording. To achieve mass spectral summing (effectively signal averaging) the irregular matrix generated by a Python script^65^ modified from a base source was used directly. The intensities in *m*/*z* bins for chosen *m*/*z* ranges are summed one spectrum at a time because the matrix is irregular. Each of the total intensities of features are multiplied by the time taken for their scans and then summed to get the time-weighted average of the total intensities of features for a selected range of spectra.

## Results and Discussion

### Mass Spectral Features

To demonstrate the superior performance of cVSSI compared with ESI, preliminary studies were performed with 36-mer triplex DNA where ion intensity was studied as a function of applied voltage. This work is described in the Supporting Information section and is summarized in Figure S2. The wide operating range of applied DC voltage and the nESI-like sensitivities obtained without source modification (Figure S2) serve as motivation for the extensive ion source characterization. As with ESI, because ion signal levels for cVSSI are greater for smaller oligonucleotide species, for the more extensive optimization studies, a slightly smaller 27-mer triplex DNA was used as the model system to simplify data analysis (e.g., comparisons of different ion species). This molecule consists of single strand (GAA)_9_ (8.538 kDa) and single strand (TTC)_9_ (8.016 kDa) nucleotide sequences to form DNA duplex. With the addition of the TFO (8.016 kDa) strand, the triplex DNA molecule has a mass of 24.570 kDa. Figure S3 in the Supporting Information section shows a schematic with the relative masses for different monomer, duplex, and triplex species that may be expected to be observed in the mass spectra. This includes triplex ions not containing cation adducts (Tri), triplex adduct ions (Tri+ad), triplex-sized fragment ions (Tri-fr), duplex ions (Dup and Dup+ad), duplex-sized fragment ions (Dup-fr), single strand ions (TFO and TFO+ad), and single strand-sized fragment ions (TFO-fr).

Although the *m*/*z* range selected for this study (*m*/*z* 1500 to 4000) encompasses that of all observed ion species across the mass ranges given above, this technical report mostly focuses on ion signal enhancement for Tri ions with special consideration given to determining the conditions/settings that preserve the noncovalent structure of the DNA with the greatest ion signal level. To achieve optimal production of the Tri ions, the total amount of undesired species such as the Tri+ad and Tri-fr must be minimized.

**Figure 1:**
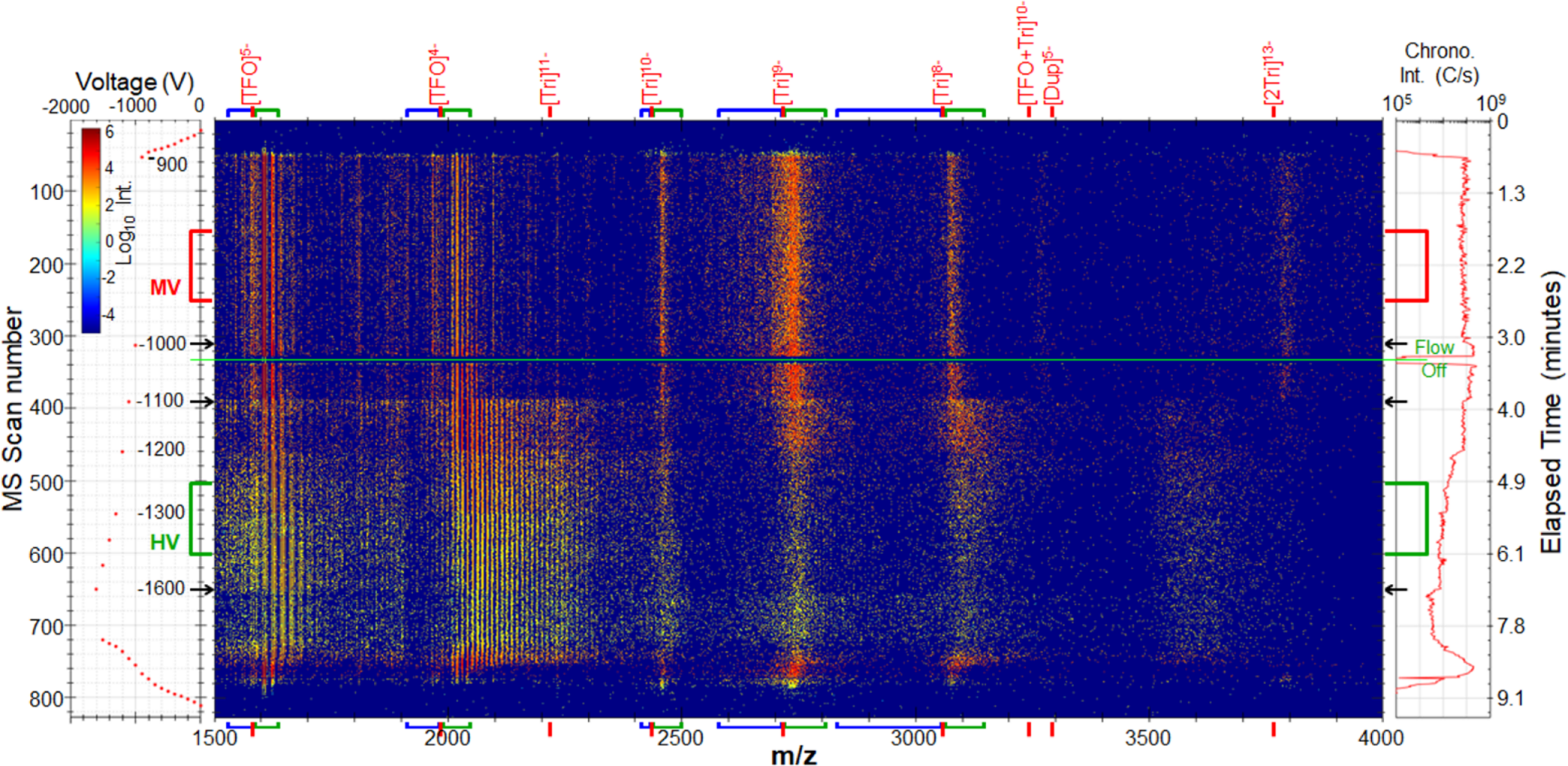
Heat map showing data set features for mass spectral scans as a function of voltage application. The left inset shows the voltage application profile beginning at zero volts. The right inset shows the ion chronogram obtained by summing ion intensities for each mass spectral scan. The different data set features are labeled as ions at the top of the heat map. The color legend shows differences in ion intensity. The inlet capillary temperature was set at 300 °C for this data set. Arrows indicate scan numbers associated with voltage changes. Red and green rectangles show medium and high-voltage regions (MV and HV, respectively) used for comparisons of ion signals (see text for details). Blue and green brackets along the *m*/*z* axis show integration regions for features associated with Tri-fr and Tri+ad ions.

Figure 1 shows features associated with DNA ions produced upon performing variable voltage cVSSI of the 27-mer DNA sample at 300 °C. Peaks corresponding to DNA ions (all kinds) are generally not observed across the voltage range of 0 to −700 V. Notably, for the 36-mer DNA ions, the highest-intensity mass spectral features for each charge state in the cVSSI mass spectrum (inset in Figure S2) were associated with Tri+ad species. Comparatively, for the 27-mer DNA triplex sample, the highest-intensity features were the Tri ions. This provided additional incentive to use the smaller molecular complex for these studies. To enhance the ion signal for DNA Tri ions, in-source collisional activation (normalized at 30 eV) was applied. This process significantly improves the analyte signal by decreasing the population of Tri+ad species. Admittedly, this ion activation setting produces some Tri-fr ions, as shown in Figure S4 and so there is a small trade-off when optimizing for the desired Tri ions. Figure S4 shows that for this oligonucleotide system effective declustering requires a collisional activation setting of 25 to 30 eV. Although ion declustering is used here, in terms of total ion abundance for a given charge state, Figure S4 shows that the adduct ions still predominate (summed together). Notably, samples were purchased with standard desalting and more extensive desalting may provide better ion production of the desired Tri species.

As observed in Figure 1, with increased voltage application to the infused sample, features corresponding to the [Tri]^8–^, [Tri]^9–^, and [Tri]^10–^ ions become evident. Also observed are those corresponding to the [TFO]^4–^ and [TFO]^5–^ ions as the most intense. A clear transition in total ion signal level is observed just after the application of −1000 V. This is evidenced by the discretized ion abundance transition in the ion chronogram in Figure 1 (first arrow) resulting in an approximate doubling in total ion intensity. Overall, this region represents the greatest summed ion intensity obtained across the voltage range studied. Another transition (second arrow in Figure 1) is observed at ∼ −1100 V corresponding to a nearly 2-fold decrease in ion signal intensity. This coincides with a greater number of features at higher *m*/*z* than those for the [Tri]^8–^, [Tri]^9–^, and [Tri]^10–^ ions as well as the [TFO]^4–^ and [TFO]^5–^ ions (Figure 1). A third transition shown in the heat map occurs at ∼ −1600 V. Overall, for most charge states, there is an increase of higher *m*/*z* DNA ions with a clear reduction of the nonadduct ion species (e.g., the clear depletion of features for the [TFO]^4–^ relative to [TFO+ad]^4–^ ions in the heat map in Figure 1). This change in relative ion abundances suggests that more of the DNA molecules acquire cation adducts. It is possible this results from increased abundance of these cation species in progeny nanodroplets^52,66^ that ultimately produce the ions at this voltage setting.

To further evaluate the voltage effects on the types of ions produced, consider Figure S5. Here, the mass spectral features associated with the [Tri]^9–^ ions are presented. At ∼ −900 V, features for the [Tri]^9–^ ions are evident. By ∼ −1000 V, features corresponding to increased adduct ions are observed where deviations in intensity are more pronounced at higher *m*/*z* values (*m*/*z* 2730 to 2760). Additionally, the mass spectral baseline in this region is higher than that of the −900 V data. This increased baseline associated with a greater overall adduct ion presence contributes to the near doubling of the total ion intensity observed just after the application of this voltage (first arrow in Figure 1). As mentioned above, for these conditions, progeny nanodroplets may have increased abundances of cation adduct species. nESI work^66^ as well as recent cVSSI work^62^ has suggested that such droplet makeup affects the types and amounts of adduct ions observed. By ∼ −1100 V, features corresponding to the [Tri]^9–^ ions are difficult to distinguish as clearly, because the [Tri+ad]^9–^ ions become the dominant species (Figure S5). This is the origin of the spreading to higher *m*/*z* of mass spectral features corresponding to different oligonucleotide ions at this voltage setting as observed in the heat map in Figure 1. As the voltage is increased above ∼ −1500 V, features associated with the [Tri]^9–^ ions are no longer distinguished in the mass spectrum (Figure S5) and those representing the [Tri+ad]^9–^ species become indistinguishable as well (broad hump in the mass spectrum). At 300 °C, the voltage range of −900 to −1000 V represents optimal conditions for production of Tri ions. Here we refer to this voltage setting as midvoltage (MV) conditions. Higher voltages (−1100 to ∼ −1500 V) are hereafter referred to as high-voltage (HV) conditions.

### Effect of Capillary Inlet Temperature on Tri Ion Production

**Figure 2:**
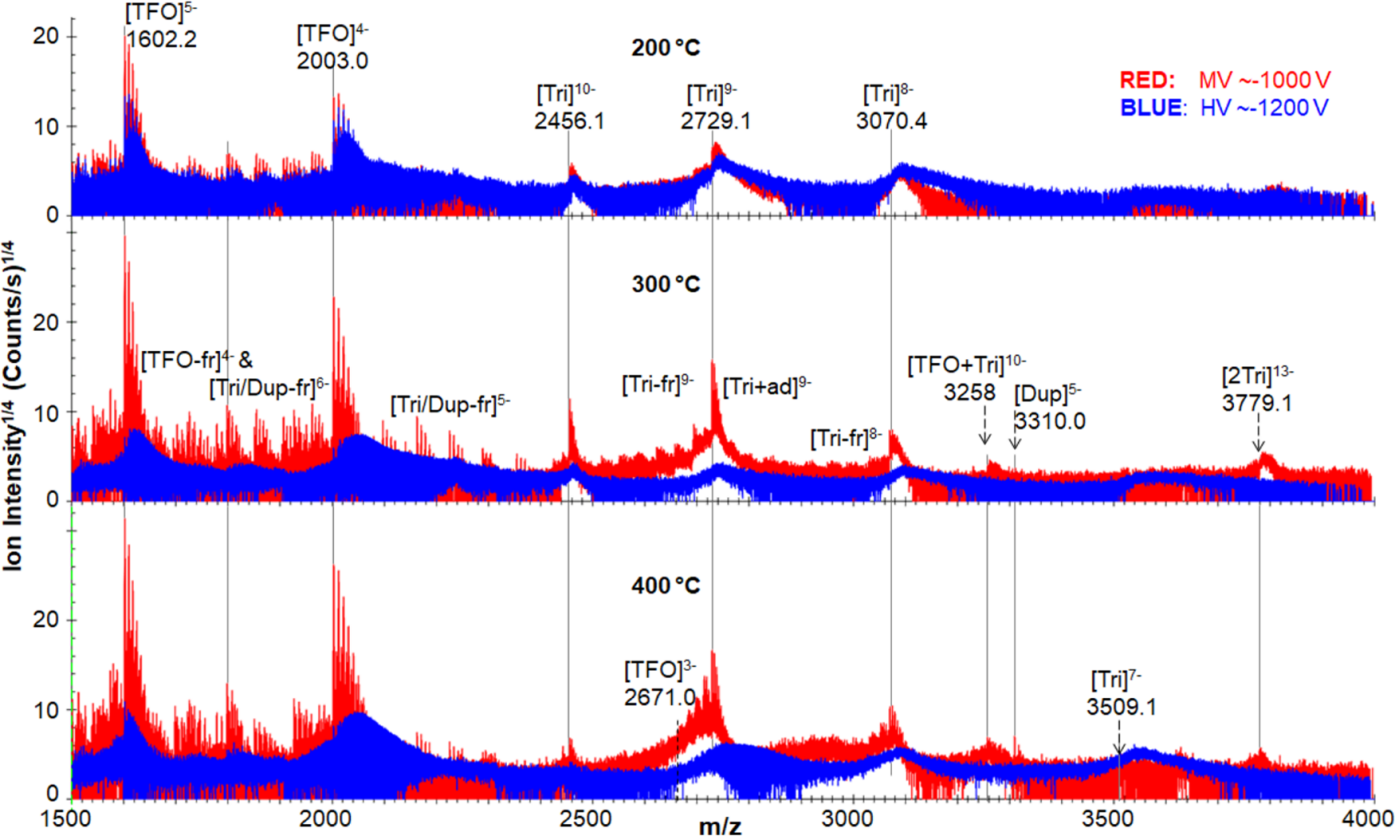
Stacked mass spectral plots showing all DNA ions at different temperatures. The top, middle, and bottom spectra represent data collected at 200, 300, and 400 °C, respectively. Red and blue traces correspond to medium and high-voltage (MV and HV) conditions, respectively (see legend for voltage values). The different ion species are labeled next to each data set feature. Vertical lines are added to guide the eyes and connect assigned features across different temperatures.

Examining the effect of voltage on the production of the desired intact Tri ions starting at 200 °C, it is possible to evaluate the ion production achieved using the MV and HV settings at different capillary inlet temperatures. Mass spectra from *m*/*z* 1500 to 4000 of the Tri system are shown in Figure 2. Note that the mass spectra in Figure 2 uses a nonlinear intensity axis to reveal changes in the lower intensity Tri+ad and Tri-fr species. Figure S6 shows the mass spectral data for the MV settings collected at 300 °C with a linear intensity scale; mass spectral features are readily assigned and the data is robust. In general, at 200 °C, the intensities of features for the TFO and Tri ions are increased in the MV range (compared to the HV range) with the exception of those associated with the [Tri]^8–^ species. Another general trend noted above is the increased amount of features for Tri+ad and TFO+ad ion species relative to those of the TFO and Tri ions under the HV settings. As the temperature increases to 300 °C, both of these trends become more pronounced. That is, the intensity improvement is greater for features of the desired Tri ions under the MV conditions relative to the HV conditions. For the MV setting the peak intensities for the [Tri]^8–^, [Tri]^9–^, and [Tri]^10–^ ions increase by ∼70, ∼260, and ∼125, respectively. Having suggested above that the increase in adduct ions results from different progeny nanodroplet composition, it appears that the progeny nanodroplets formed under HV conditions may become enriched in these cation species at higher temperatures. It is interesting to consider that the more rapid desolvation of the large droplets may lead to less loss of cationic species by ion evaporation events thereby contributing to increased adduct ion production. Additionally, some features for Tri-fr, and Dup-fr ions are observed under the MV conditions. Finally, the largest features for the Tri and Tri+ad ion clusters shift slightly further to higher *m*/*z* for the HV conditions at 300 °C indicating the increase of the latter species; indeed, the ratio of Tri to Tri+ad ions is decreased ∼6 fold. Increasing the capillary temperature further to 400 °C results in increased broadening of the adduct ion peaks observed at HV conditions. For the MV conditions at this temperature, ion fragmentation is especially noticeable with the increased presence of features for the Tri-fr species (Figure 2).

**Figure 3:**
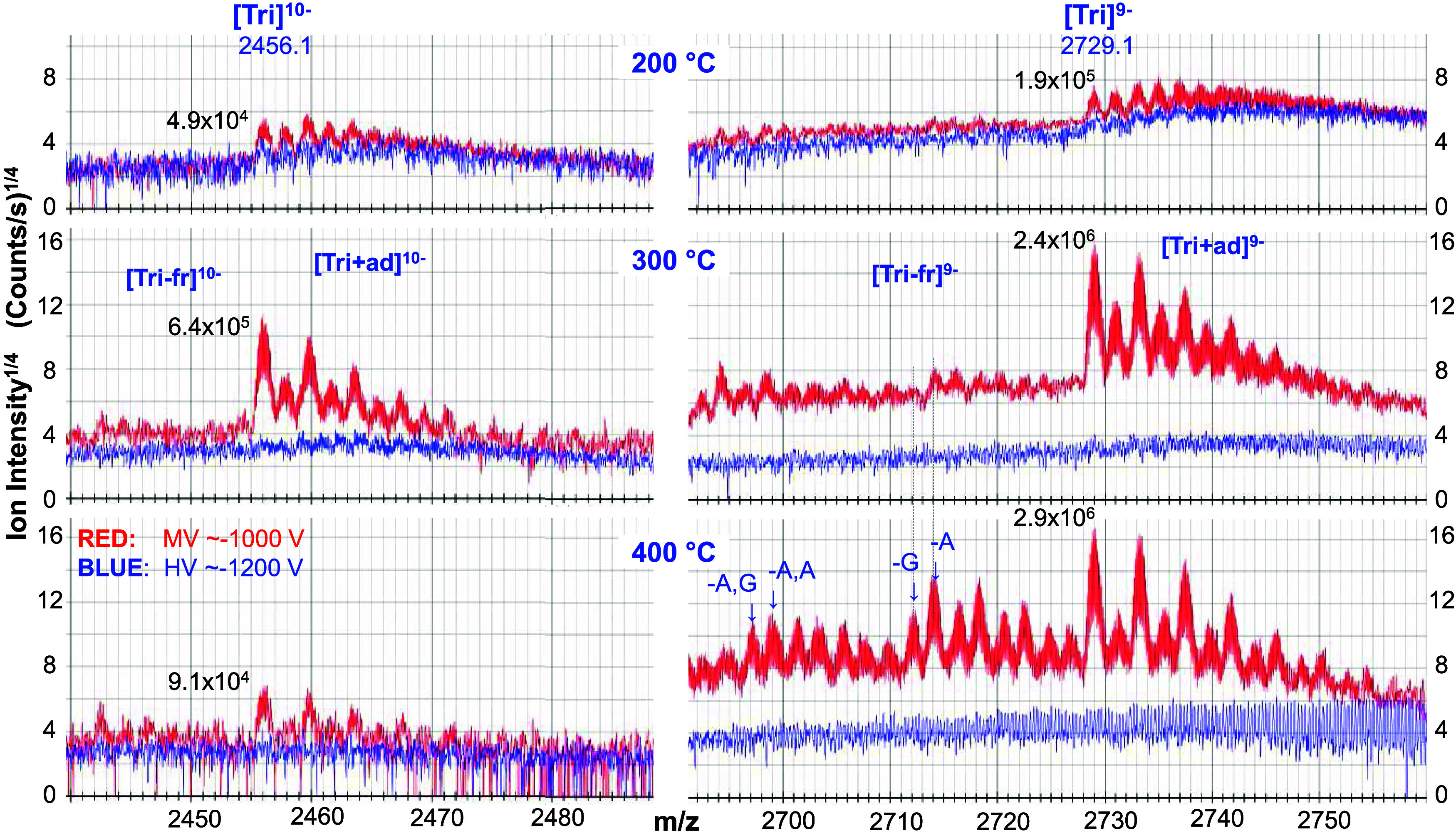
Stacked plots of zoomed-in mass spectral regions for Tri ions. The left and right panels show results for 10- and 9- ions, respectively. The mass spectral ranges encompass major Tri-fr, Tri, and Tri+ad ions and these are labeled for each charge state. Mass spectra at different capillary inlet temperatures are provided and the temperature is labeled. The peak intensities for the [Tri]^10–^ and [Tri]^9–^ ions are provided where observed. Note that the intensities are plotted on y^1/4^ scale in order to emphasize the abundances and types of Tri-fr and Tri+ad ions produced. For these data sets, the resolving power of the mass spectrometer was set at 140,000. Several ion fragments indicating the loss of bases A and G are labeled.

For a more detailed comparison of Tri-fr, Tri, and Tri+ad species produced as a function of temperature, consider Figure 3 which shows the zoomed-in mass spectral regions encompassing features for the [Tri]^10-^ and [Tri]^9–^ ions at different heated inlet transfer tube temperatures (Figure S7 shows the data for all temperatures). Figure 3 shows that the peaks associated with the [Tri]^10–^ and [Tri]^9–^ ions are larger when using the MV settings at all three temperatures. Comparatively, features corresponding to Tri ions are not observed at 150 °C for either MV or HV settings (Figure S7). Under HV settings, features of the Tri ions become indistinguishable by 300 °C. The lack of detectable peaks under both voltage settings at 150 °C, can be attributed to inefficient ion desolvation for the very large droplets produced by cVSSI.^60,62^ The loss of distinguishable ions for the HV settings at higher temperatures can be attributed to the production of a much greater amount of Tri+ad species (greater ion heterogeneity) as well as interfering TFO^3–^ ions (See Figure 2).

The data presented in Figures 2 and 3 suggest that the HV conditions are not well suited for analysis of large DNA biopolymers with cVSSI-native MS. Hereafter, the discussion focuses on the results for the MV conditions. First consider the peak intensities associated with the Tri ions. When the heated inlet transfer tube temperature is set to 200 °C, the features for [Tri]^10–^ and [Tri]^9–^ ions begin to appear as shown in Figure 3. For example, the peak abundances associated with the [Tri]^10–^ and [Tri]^9–^ ions are increased by ∼13-fold each when the heated inlet transfer tube temperature is set to 300 °C compared with 200 °C. When the temperature is set at 400 °C, the [Tri]^10–^ ion intensity decreases by 7 fold while that of the [Tri]^9–^ ion increases slightly. At 450 °C, the former ions are not observed while the intensity level of the latter decreases by an order of magnitude (see Figure S7).

Additional mass spectral feature changes among the data sets represented in Figure 3 are observed. At 200 °C, some of the Tri+ad features are more intense than those of Tri ions for both charge states. By 300 °C, this is no longer evidenced as the Tri ion features are the dominant peaks for both charge states. Presumably this results from increased ion activation occurring in the source region. Coincident with the decrease in Tri+ad ion peaks at 300 °C, is the observation of Tri-fr ion peaks for both charge states (see Figure 3) indicating that greater ion fragmentation is observed at the higher temperature. Indeed, by 450 °C, the intensities of some [Tri-fr]^9–^ ion peaks are greater than those of [Tri]^9–^ ions (see Figure S7). This temperature dependence is also observed for the [Tri]^10–^ ion peaks; however, these peaks are not observed at the highest temperature setting. The Tri-fr peaks shown in Figure 3 represent triplex-sized fragments which involve the loss of one or more bases from the Tri and Tri+ad precursor ions. Because the focus of this work is the optimization of cVSSI for native MS, the exact structure and assignment of only some of these ions is presented in Figure 3. A detailed description of the origin of these fragment ions and their structures will be explored in future studies using in-depth examination of the Tri and Tri+ad clusters as fragment ion patterns/templates associated with Tri species. Here, such ions are treated as undesirable insofar as they deplete the Tri ion signal levels.

### Evaluating cVSSI Source Performance in the MV Regime

Tuning the cVSSI method necessarily involves multiple steps such as the formation of a robust and continuous plume of microdroplets containing the analytes, applying the appropriate DC voltage to the liquid sample, and utilizing the best heated inlet transfer tube temperature for the plume and droplet charge conditions. For these studies, the same emitter tip was used for all experiments to reduce the effect of droplet plume variability (largely initial droplet size). This allows us to focus on the applied voltage and capillary inlet temperature. It is noted that prior studies have shown that tip aging effects are minimal over these tens of minutes experiments;^62^ additionally, in-droplet hydrogen–deuterium exchange (HDX) studies that employ two cVSSI emitter tips have shown that tip-to-tip plume reproducibility is extremely robust.^37^ As presented in the discussion of Figures 1–3, the ideal voltage for ionization by cVSSI of large DNA biopolymers is suggested to be −900 to −1000 V. This corresponds with a field of 4.5 to 5.0 × 10^3^ V/cm. To determine the optimal capillary temperature, it is useful to plot the abundances of the desired Tri ions as well as the undesired Tri+ad, Tri-fr and TFO ions as a function of temperature using the optimized MV settings. Here TFO ions are considered undesirable as any increase in such species could result from activation of Tri ions.

**Figure 4:**
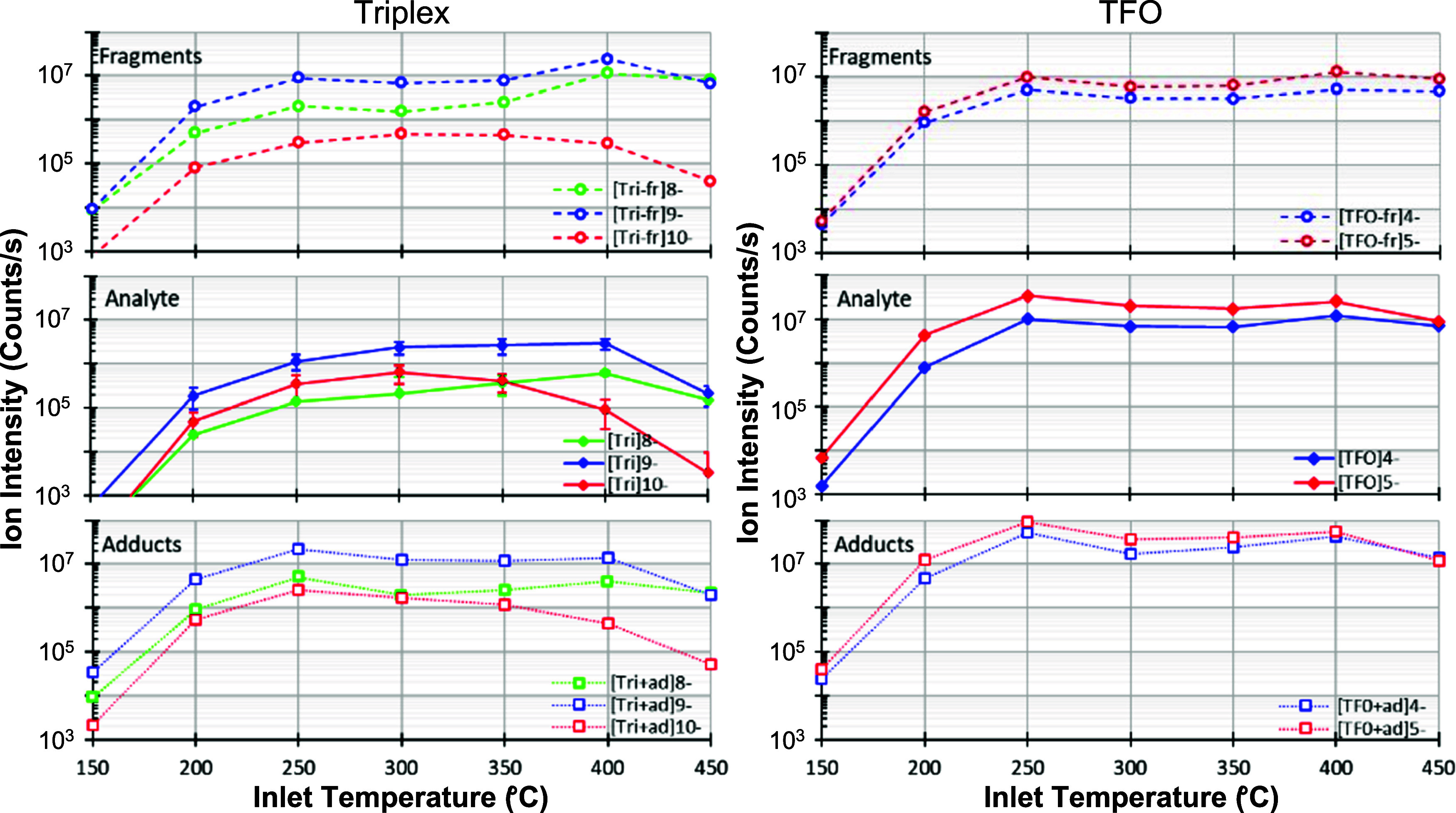
Ion intensity plots as a function of temperature for major Tri and TFO ion species. Data for Tri and TFO DNA are shown on the left and right as labeled in the figure. Legends provide the ion identities. The *y*-axis is represented on a logarithmic scale for facile comparison across all capillary inlet temperature settings. Lines are provided to guide the eyes in the comparison of different ions. Error bars (middle panel on the left) show the scan-to-scan variability of Tri ion intensities across the voltage range.

Figure 4 shows that the [Tri]^10–^ ions are most abundant at 300 °C while the [Tri]^9–^ and [Tri]^8–^ ion abundances maximize at 400 °C. By 450 °C, all charge states have decreased dramatically. The [Tri]^10–^ ion abundance decreases by more than 2 orders of magnitude from its maximal value. In comparison, Tri+ad species exhibit maximum abundances at a lower temperature of 250 °C and decrease at higher temperatures. This is again suggestive of in-source ion declustering due to greater ion activation at elevated temperatures (see Figure S4). The Tri-fr curve in Figure 4 shows an initial ion abundance plateau commencing at 250 °C and extending to 350 °C before experiencing a noticeable increase at 400 °C for [Tri-fr]^9–^ and [Tri-fr]^8–^ ions. The [Tri-fr]^10–^ ion abundance decreases in this range but this may be attributed to decreased ion production of this charge state as a whole at higher temperatures. Noticeably, the ion abundances for the Tri+ad, Tri, Tri-fr species having the *m*/*z* values used in the integration step are all decreased at 450 °C. This may result because of further ion fragmentation in the source at this high temperature to produce a greater heterogeneity of Tri-fr species that occupy a wider range of *m*/*z* values in the mass spectrum and that are not fully resolved and/or detected in sufficient abundance. Considering the scan-to-scan variability in the Tri ion intensities (Figure 4), the different charge states exhibit nearly the same intensity from 300 to 400 °C. However, the larger amount of adducts at the lower temperature and the increased fragmentation at the higher temperature suggests best Tri ion production occurs between 300 to 350 °C.

TFO+ad, TFO, and TFO-fr ion production as a function of temperature under the MV settings show trends that are remarkably similar as demonstrated in Figure 4. First, each of these species exhibits a local maximum abundance value at 250 °C. Then the ion abundances of these species drop slightly up to 400 °C. Here, they again increase. Finally, the abundances of all these ions decrease when the temperature is set to 450 °C. It is noted that this reduction in ion abundance is not as great as that observed for the Tri ions.

### Effects of the Heated Inlet Transfer Tube on Ion Formation Mechanism and Fragmentation

Multiple studies have shown that for ESI of small molecules, ionization efficiency best correlates with the base dissociation constant, *K*_b_.^67,68^ For native MS, it has been argued that preformed biopolymer ions in solution are merely transferred into the gas-phase via the charge residue model (CRM).^69^ More recently, the chain ejection model (CEM) has been described^70,71^ and argued to be responsible for the production of ions from unfolded and partially folded species in solution.^72−74^ Here, biopolymer ions are formed as charge migration occurs onto a portion of the biopolymer protruding from the droplet due to Coulomb repulsion at the nanodroplet surface. Thus, it can be argued that the delicate balance between droplet surface Coulomb repulsion and nascent gas-phase ion charge stabilization control ion production to a greater extent than *K*_b_. A question arises as to whether the Tri ions are produced via the CRM or CEM mechanism. The relatively narrow and low charge state distribution would argue for the former. That said, the production of some ions via the CEM cannot be ruled out with the present experiments.

Using the assumption of ion production primarily via CRM, the changes in mass spectral peaks associated with different ions can be explained considering heated inlet transfer tube temperature effects on this mechanism. It has long been postulated^75^ and widely suggested^76^ that the heated inlet transfer tube aids droplet and/or ion desolvation for ESI. Indeed, many studies are dedicated to improving atmospheric droplet desolvation for such sources.^77^ Thus, for our studies, where cVSSI produces large aqueous droplets, at low heated inlet transfer tube temperatures (150 and 200 °C), lower peak intensities are likely associated with decreased droplet and/or ion desolvation leading to species removal by differential pumping and/or increased ion heterogeneity and/or size such as not to be detected by the mass spectrometer. Higher temperatures (250 to 400 °C) lead to increased ion abundances of all ions indicating improved solvent evaporation and faster production of the gas-phase ions via the CRM. This implies that more heat is absorbed by the droplets during transit in the capillary. Interestingly, the abundances of Tri-fr ions increase dramatically at 400 °C (Figure 4). This further supports the hypothesis that the droplets become more heated. That is, as the conditions in the source are already activating to a degree (Figure S4), a small change in the internal energies of the emerging ions from droplet heating coupled with increased collision gas temperatures result in a shift to Tri-fr ion production. Finally, at the highest temperatures (450 °C), the decrease in most ion species can be attributed to even more ion fragmentation. Here, the Tri-fr heterogeneity increases and their associated signals expand to *m*/*z* regions not included in the integration employed for the data shown in Figure 4. Therefore, it is not possible to account for such species as they are not of sufficient intensity or resolved; rather, they are represented by many peaks dispersed throughout the mass spectrum. Because different ion sources produce different ion activation conditions^78^ and employ different heated capillaries, the results described here will not be standardized for all systems. However, the work lays the foundation for developing a means to rapidly determine optimal settings on any mass spectrometer by revealing relative field and heating effects based on mass spectral comparisons.

## Conclusions

This study presents the first optimization of the novel cVSSI source for native MS experiments of large oligonucleotides. Overall, for traditional cVSSI emitter tips (typically ∼20 μm ID) operated with a solution flow rate of 2 μL/min and positioned ∼ 2 mm from the mass spectrometer inlet, the optimal field strength is 4.5 to 5.0 × 10^3^ V/cm. Under the heated inlet transfer tube temperatures examined here, this field strength provided the greatest ionization efficiency for Tri ions (reduced Tri+ad and Tri-fr ions). This voltage setting when coupled with modest in-source ion declustering provided the greatest *S*/*N* ratios for Tri ion peaks in the mass spectra. The heated inlet transfer tube temperature settings demonstrated that the best operational conditions for large oligonucleotide systems are 300 to 350 °C. These settings avoid excessive production of Tri+ad ions at lower temperatures and Tri-fr ions at higher temperatures. Although there are many other parameters to be explored for this new ionization source (e.g., initial droplet size), given that the methodology already provides a remarkable ion signal advantage over ESI (Figure S2) and the corona discharge workaround, such studies as those presented here already enable researchers to perform more efficient native MS experiments for large oligonucleotide species using cVSSI. Future work will focus on the effects of other buffer systems such as triethyl and trimethylammonium acetate as these have been demonstrated useful in the MS study of oligonucleotides.^37,79^ Additionally, as the studies here have revealed the delicate balance between ion declustering and ion fragmentation in the source as influenced by heated inlet transfer tube temperature (Figures 3 and S4), a careful mapping of this parameter space for each buffer system will be required as well. Finally, because the field strength and heated inlet transfer tube temperature are linked to phenomena such as the progeny nanodroplet composition and ion formation mechanism, it is expected that these findings are relevant for other biopolymers examined in negative-ion mode. As mentioned herein, multiple studies have suggested that negative-ion mode should be employed for different acidic biopolymers.^80−83^ Therefore, in the future the studies will be extended to the analysis of native structure of some other biologically important and therapeutically relevant oligonucleotides such as 10–23 DNAzyme, ribozyme, i-motifs, and mRNA systems.

## Supporting Information Available

Short description of motivation for using cVSSI and its advantages; schematic diagrams of the experimental setup and the molecular system studied; ion intensity vs voltage plot showing the advantage of VSSI; zoomed-in mass spectra showing the effects of in-source CID settings; ion transfer tube temperature and applied voltage (PDF)

## Notes

^§^ S.M. and V.K.D. contributed equally to this work.
