# Supplementary material for: Optimization of Capillary Vibrating Sharp-Edge Spray Ionization for Native Mass Spectrometry of Triplex DNA
Source: ACS Omega. 2025 Mar 27;10(13):13131–40. doi: 10.1021/acsomega.4c10615 (PMC11983206; doi:10.1021/acsomega.4c10615)
Supplement: Supplementary file 1 — ao4c10615_si_001.pdf [file ao4c10615_si_001.pdf]

Optimization of capillary vibrating sharp-edge spray ionization for native mass spectrometry of triplex DNA

Sultan Mahmud,[1ǂ] Vikum K. Dewasurendra,[2ǂ] Chandrima Banerjee,[1] Pedram Tavadze,[2] Mst Nigar Sultana,[1] Mohammad Rahman,[1] Shohag Ahmed,[1] Peng Li,[1] Matthew B. Johnson,[2*] and Stephen J. Valentine[1*]

[1]Department of Chemistry, West Virginia University, Morgantown, WV 26506
[2]Department of Physics and Astronomy, West Virginia University, Morgantown, WV 26506
ǂAuthors contributed equally
*stephen.valentine@mail.wvu.edu, matthew.johnson@mail.wvu.edu

Supporting Information

Demonstration of cVSSI for 36mer DNA triplex 2
Figure S1 4
Figure S2 5
Figure S3 6
Figure S4 7
Figure S5 8
Figure S6 9
Figure S7 10
References 11

Motivation for using cVSSI in native MS of oligonucleotide species. In the past, it has been argued that native MS of oligonucleotides is best performed in negative ion mode.[1] Additionally, it has been argued that many acidic biopolymers may ionize more efficiently in negative ion mode.[2-4] To demonstrate the utility of cVSSI in the performance of native MS of large oligonucleotides, preliminary experiments examined the production of 36-mer DNA triplex ions as a function of tip-inlet voltage. As mentioned in the article, DNA triplex (Tri) consists of three single stranded DNA sequences. For the 36-mer triplex, single strand $(GAA)_{12}$ (11.405 kDa) and single strand $(TTC)_{12}$ (10.708 kDa) nucleotide sequences comprise the duplex. The additional single strand $(TTC)_{12}$ represents the triplex forming oligonucleotide (TFO, 10.708 kDa) which is required for the formation of 36-mer triplex DNA (32.821 kDa).

To demonstrate the improvement of oligonucleotide ionization in negative ion mode by cVSSI relative to ESI, a single emitter tip was utilized. Ion signals were examined with and without the use of emitter tip vibration. Here the flow rate was maintained at 2 µL/min. Thus, the comparison represents that of ESI in the very low microflow regime. Here microflow analysis is performed because when comparing to cVSSI, the capillary action resulting from the mechanically nebulized sample makes it difficult to establish a flow rate below 1 µL/min using these cVSSI emitter tips. Admittedly, nESI experiments could produce greater ionization efficiency than ESI conducted with the same emitter tips in the very low microflow regime. However, the working range (voltage range over which ions are produced) would still be very limited by the corona discharge problem.[5] Additionally, the microflow regime is better suited to robust hyphenated techniques with regard to experimental reproducibility (spray stability and tip clogging) and throughput (changing samples).[6] It can be argued that here many of the attendant problems of nESI are removed with the application of higher flow cVSSI including the preservation of fragile biopolymer structure without sacrificing ion signal.[7]

Figure S2 shows the ion intensities for a sample of 36-mer triplex DNA obtained from mass spectra collected over a voltage range of -500 V to -1500 V. Notably, at the voltage associated with the greatest ion production by ESI, a nearly 60-fold advantage in ion signal level for the most abundant $[M-nH]^{n-}$ ions is achieved with cVSSI. At the peak voltage for cVSSI this advantage extends to above 100 fold (see Figure S2). The operational range of applied voltage resulting in ion production is much broader for cVSSI as well (~700 V compared with ~200 V – see Figure S2).

The signal-averaged mass spectrum extracted at the optimal voltage setting for ESI exhibits a remarkable difference compared to that obtained for cVSSI as shown in Figure

S2. Visual inspection of the mass spectra reveals that the proportion of adduct ions species (Na^+, K^+, and possibly NH_4^+ and combinations) is much greater for the ESI data compared with that of cVSSI. This leads to considerable degradation in the overall signal-to-noise (S/N) ratio of the analyte ion peaks observed. Here it is noted that the study described in the text is performed to determine the optimal conditions to extend this ionization advantage to a maximum.

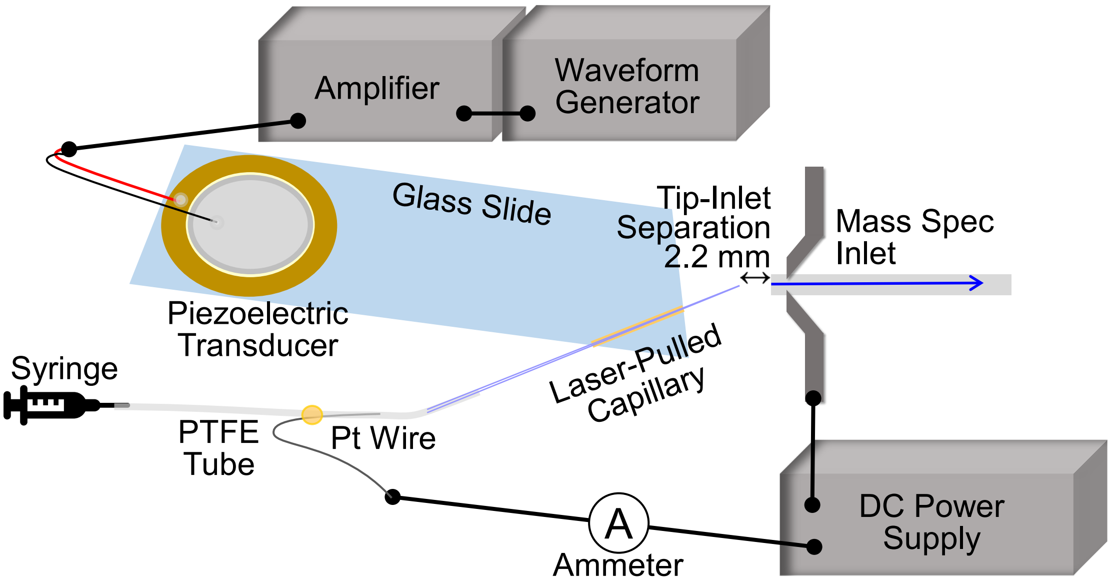

Figure S1. Schematic of cVSSI device. Shown are the RF and DC power supplies, waveform generator, and amplifier required to perform cVSSI experiments. Also shown are the cVSSI device component parts. Note that DC voltage is applied to sample solution immediately preceding the pulled capillary tip. See text for details. Note that component parts are not drawn to scale.

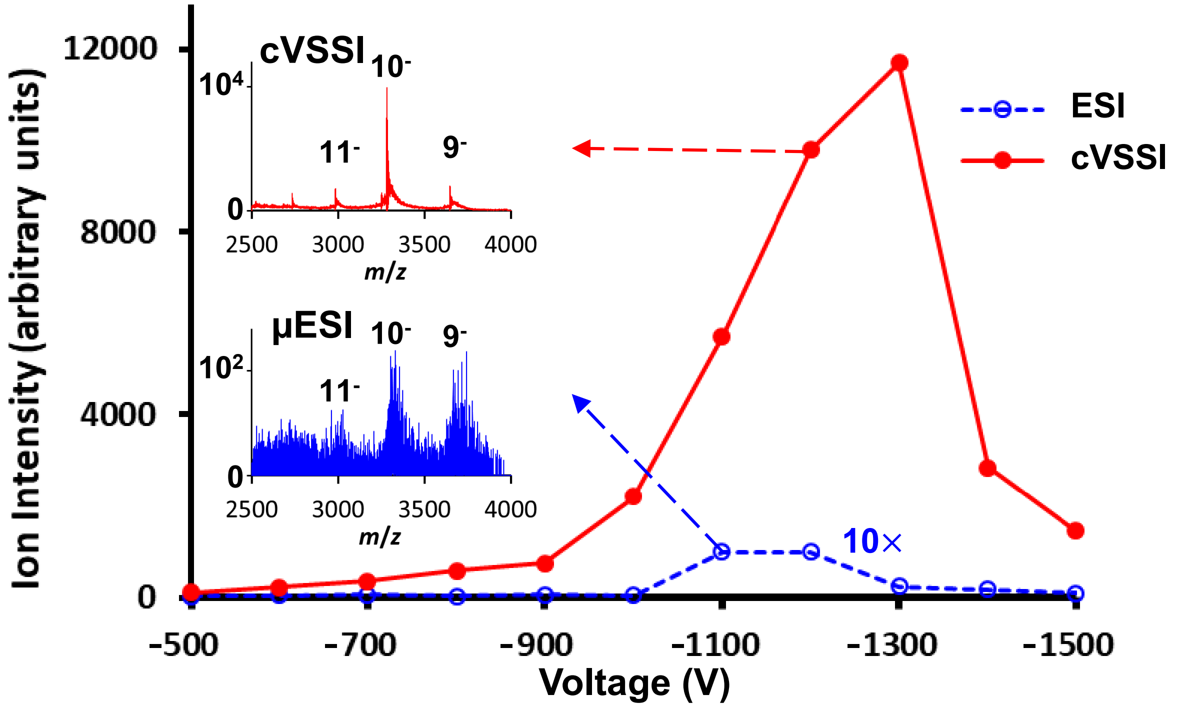

Figure S2. Ion intensities for 36mer Tri ions as a function of applied voltage. The red and blue traces correspond to cVSSI and μESI experiments, respectively. The inset mass spectra are the signal averaged spectra collected at the voltages indicated by the arrows on the x-axis.. The charge states for each ion are labeled in the inset.

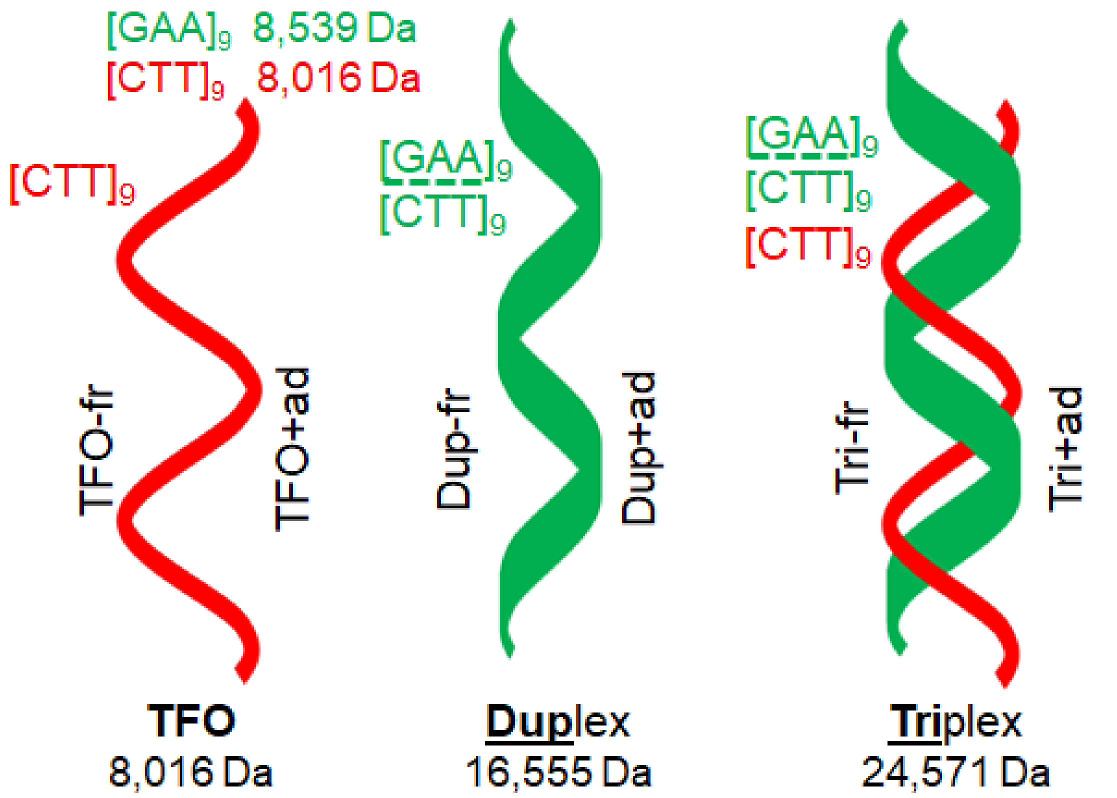

Figure S3. Cartoon representing the molecular ensemble that may be present in solution for the 27-mer DNA triplex sample. Masses are provided for intact species. Also shown are the locations of other ion species observed in the different spectra. The oligonucleotide sequences comprising each molecular species are provided above. The ion abbreviations used throughout the text are provided in bold letters below the corresponding mass.

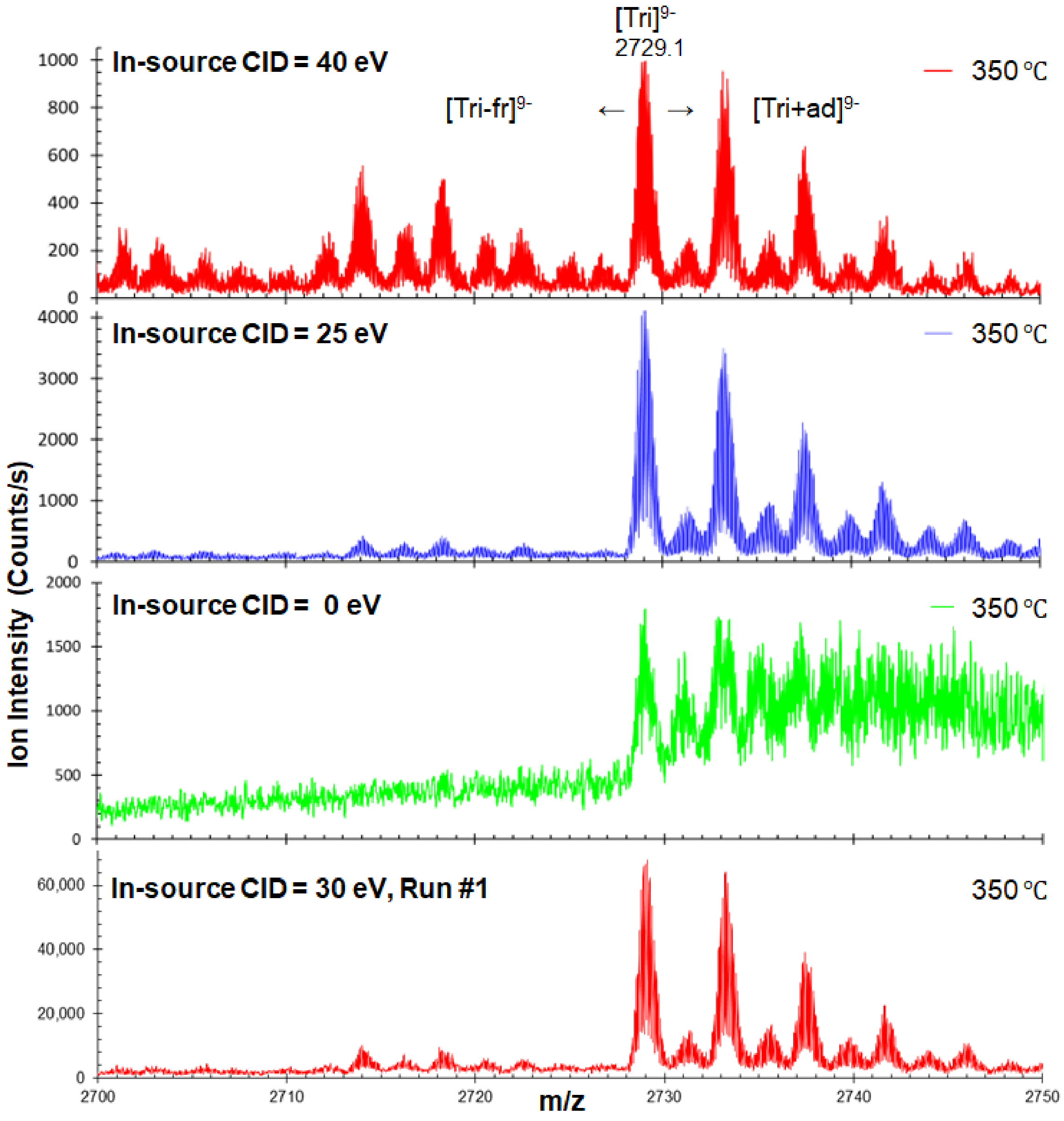

Figure S4. Stacked plots of the zoomed-in mass spectral regions of the [Tri-fr]$^{9-}$, [Tri]$^{9-}$, and [Tri+ad]$^{9-}$ species. The top three panels present data collected using a single VSSI device. The mass spectral traces from top to bottom represent decreasing values of in-source ion activation employed as labeled. The bottom panel shows data collected with the VSSI device used to collect the temperature-dependent data. These data were collected using a voltage of ~-1000 V and flow rate of 6 μL/min. Also provided are the ion intensity values. The resolving power employed for these data was 140,000. The different ion regions are labeled. The signal averaged mass spectral traces are provided.

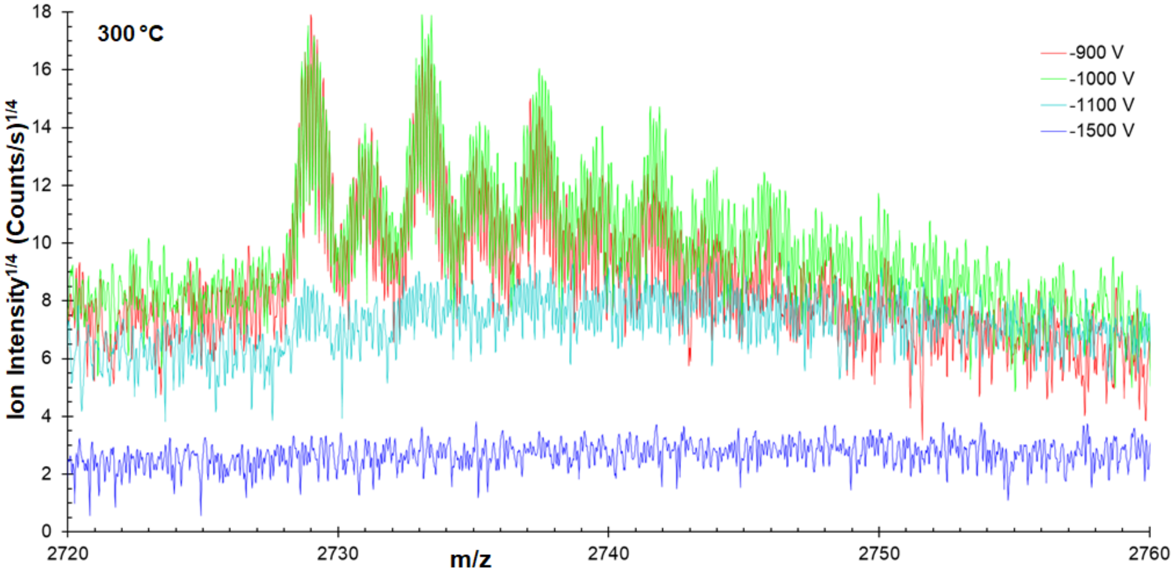

Figure S5. Overlayed, zoomed-in regions of mass spectra for the $[Tri\text{-}fr]^{9-}$, $[Tri]^{9-}$, $[Tri{+}ad]^{9-}$ ions at different voltages. These data were collected using a capillary inlet temperature setting of 300 °C. The legend provides the voltage color code for the datasets. Each scan has 20 scans averaged except for 900 V, which has 13 scans averaged. A resolving power of 140,000 was used to collect these data. For the relationship between each voltage setting and the total ion intensity, see Figure 2 and the discussion in the text. Intensities are shown on a $y^{1/4}$ scale to enhance comparisons of lower abundance ions such as $[Tri{+}ad]^{9-}$ species.

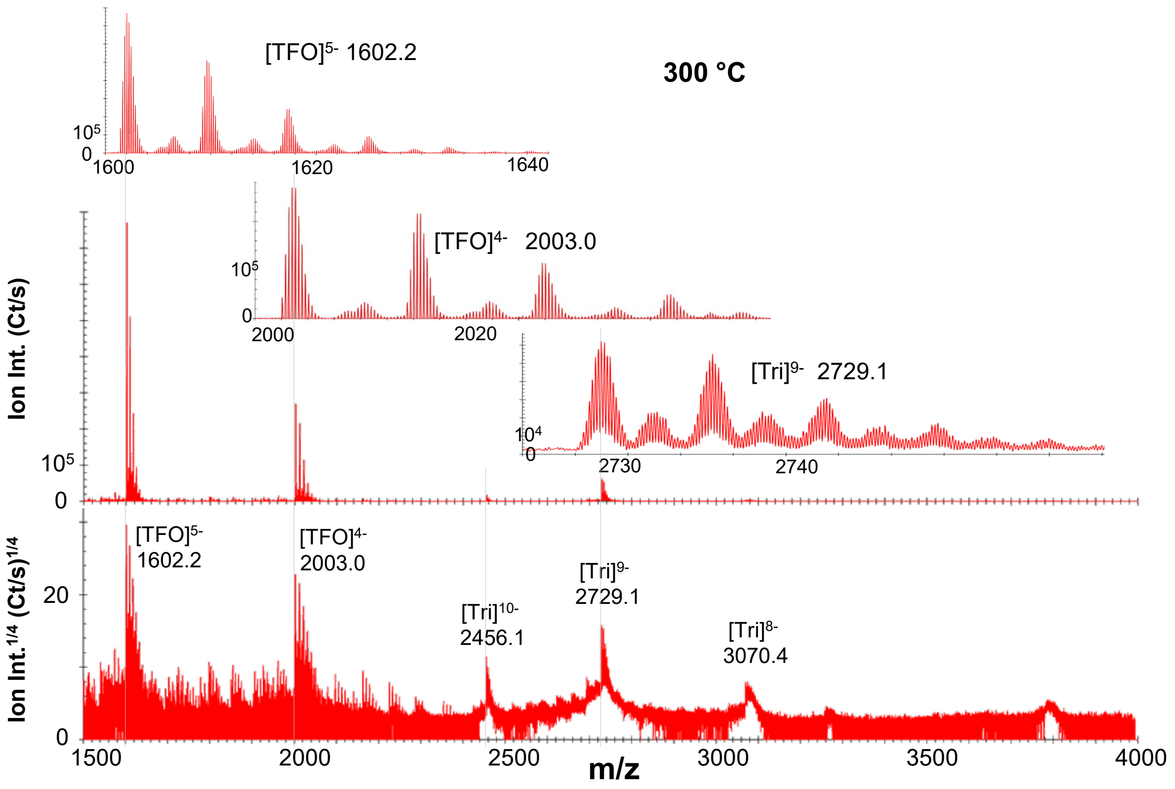

Figure S6. Mass spectrum of the 300 °C data collected under MV conditions. The bottom trace shows the data plotted on an $y^{1/4}$ intensity scale while the top trace shows the data plotted on a linear scale. The insets show zoomed-in regions of assigned peaks that are plotted on a linear scale to reveal the isotopic distributions of the different peaks.

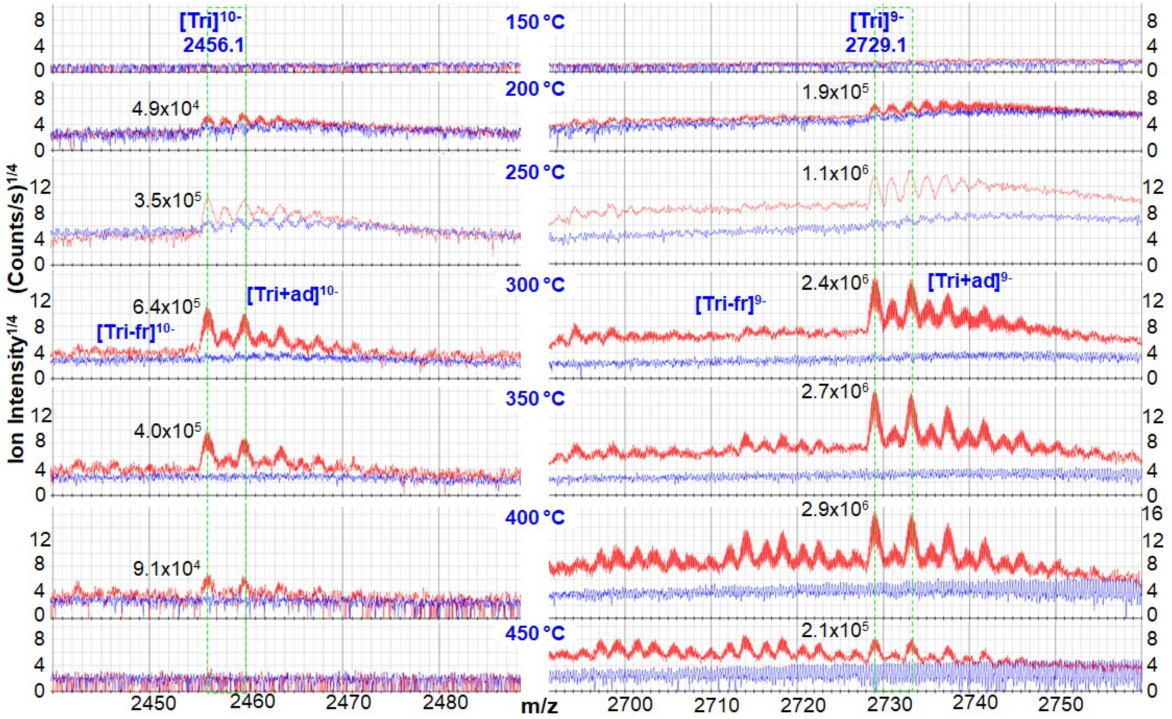

Figure S7. Stacked plots of zoomed-in mass spectral regions for Tri ions. The left and right panels show results for 10- and 9- ions, respectively. The mass spectral ranges encompass major Tri-fr, Tri, and Tri+ad ions and these are labeled for each charge state. Mass spectra at different capillary inlet temperatures are provided and the temperature is labeled. The ion abundance for the $[Tri]^{10-}$ and $[Tri]^{9-}$ ions is provided where observed. Note that the intensities are plotted on $y^{1/4}$ scale in order to emphasize the abundances and types of Tri-fr and Tri+ad ions produced. For these datasets, the resolving power of the mass spectrometer was set at 140,000 with the exception of the 250 °C traces (first dataset collected) that employed a resolving power of 70,000.

References

(1) Rosu, F.; Pirotte, S.; Pauw, E. D.; Gabelica, V. Positive and negative ion mode ESI-MS and MS/MS for studying drug–DNA complexes. *International Journal of Mass Spectrometry* **2006**, *253* (3), 156-171. DOI: https://doi.org/10.1016/j.ijms.2005.11.027.

(2) Ganisl, B.; Taucher, M.; Riml, C.; Breuker, K. Charge as you like! Efficient manipulation of negative ion net charge in electrospray ionization of proteins and nucleic acids. *European Journal of Mass Spectrometry* **2011**, *17* (4), 333-343. DOI: 10.1255/ejms.1140.

(3) Pan, P.; Gunawardena, H. P.; Xia, Y.; McLuckey, S. A. Nanoelectrospray ionization of protein mixtures:: Solution pH and protein p<i>I</i>. *Analytical Chemistry* **2004**, *76* (4), 1165-1174. DOI: 10.1021/ac035209k.

(4) Penanes, P. A.; Gorshkov, V.; Ivanov, M. V.; Gorshkov, M. V.; Kjeldsen, F. Potential of Negative-Ion-Mode Proteomics: An MS1-Only Approach. *J Proteome Res* **2023**, *22* (8), 2734-2742. DOI: 10.1021/acs.jproteome.3c00307 From NLM.

(5) Kebarle, P.; Verkerk, U. H. ELECTROSPRAY: FROM IONS IN SOLUTION TO IONS IN THE GAS PHASE, WHAT WE KNOW NOW. *Mass Spectrometry Reviews* **2009**, *28* (6), 898-917. DOI: 10.1002/mas.20247.

(6) Bian, Y.; Zheng, R.; Bayer, F. P.; Wong, C.; Chang, Y. C.; Meng, C.; Zolg, D. P.; Reinecke, M.; Zecha, J.; Wiechmann, S.; et al. Robust, reproducible and quantitative analysis of thousands of proteomes by micro-flow LC-MS/MS. *Nat Commun* **2020**, *11* (1), 157. DOI: 10.1038/s41467-019-13973-x From NLM.

(7) Sharif, D.; Foroushani, S. H.; Attanayake, K.; Dewasurendra, V. K.; DeBastiani, A.; DeVor, A.; Johnson, M. B.; Li, P.; Valentine, S. J. Capillary Vibrating Sharp-Edge Spray Ionization Augments Field-Free Ionization Techniques to Promote Conformer Preservation in the Gas-Phase for Intractable Biomolecular Ions. *The Journal of Physical Chemistry B* **2022**, *126* (44), 8970-8984. DOI: 10.1021/acs.jpcb.2c04960.
